# Supplementary material for: Random sampling causes the low reproducibility of rare eukaryotic OTUs in Illumina COI metabarcoding
Source: PeerJ. 2017 Mar 22;5:e3006. doi: 10.7717/peerj.3006 (PMC5364921; doi:10.7717/peerj.3006)
Supplement: Table S1 — Specimens are ranked based on the proportion of sequences recovered in the metabarcoding dataset. [file peerj-05-3006-s001.docx]

| Specimen no. | Phylum | Subclass/Order | Family | Genus/Species | Rank | GenBank accession no. |
| --- | --- | --- | --- | --- | --- | --- |
| FTP_0008 | Arthropoda | Decapoda | Alpheidae | Alpheus | 32 | KP254114 |
| FTP_0025 | Arthropoda | Decapoda | Panopeidae | Panopeus occidentalis | 15 | KP254222 |
| FTP_0046 | Arthropoda | Decapoda | Alpheidae | Alpheus | 8 | KP255097 |
| FTP_0048 | Platyhelminthes |  |  |  | 27 | KP254748 |
| FTP_0050 | Annelida | Phyllodocida | Hesionidae |  | 9 | KP255278 |
| FTP_0063 | Chordata | Batrachoidiformes | Batrachoididae | Opsanus tau | 2 | KP254100 |
| FTP_0077 | Mollusca | Sacoglossa | Plakobranchidae | Elysia subornata | 17 | KP255000 |
| FTP_0081 | Mollusca |  |  |  | 29 | KP254059 |
| FTP_0090 | Annelida |  |  |  | 12 | KP254797 |
| FTP_0125 | Annelida | Phyllodocida | Polynoidae | Harmothoe longidentis | 20 | KP254266 |
| FTP_0138 | Echinodermata | Ophiurida |  |  | 6 | KP254207 |
| FTP_0207 | Platyhelminthes |  |  |  | 34 | KP254368 |
| FTP_0209 | Annelida |  |  |  | 1 | KP255183 |
| FTP_0219 | Arthropoda | Decapoda | Portunidae | Charybdis hellerii | 24 | KP255147 |
| FTP_0221 | Arthropoda | Mysida | Mysidae |  | 30 | KP255166 |
| FTP_0226 | Arthropoda | Decapoda | Panopeidae | Dyspanopeus sayi | 16 | KP254742 |
| FTP_0242 | Annelida | Amphinomida | Amphinomidae | Eurythoe complanata | 23 | KP255001 |
| FTP_0248 | Arthropoda | Decapoda | Xanthidae | Menippe mercenaria | 11 | KP254868 |
| FTP_0249 | Arthropoda | Decapoda | Porcellanidae | Petrolisthes armatus | 22 | KP255241 |
| FTP_0254 | Arthropoda | Decapoda | Alpheidae | Alpheus | 25 | KP254357 |
| FTP_0262 | Arthropoda | Decapoda | Alpheidae | Synalpheus hemphilli | 26 | KP254843 |
| FTP_0264 | Arthropoda | Decapoda | Majidae | Microphrys bicornutus | 10 | KP254259 |
| FTP_0277 | Chordata | Perciformes | Gobiinae | Coryphopterus glaucofraenum | 13 | KP253993 |
| FTP_0279 | Chordata | Perciformes | Blennidae |  | 4 | KP254117 |
| FTP_0281 | Arthropoda | Decapoda | Alpheidae |  | 31 | KP254531 |
| FTP_0282 | Arthropoda | Decapoda | Majidae | Mithraculus forceps | 18 | KP254745 |
| FTP_0286 | Annelida | Phyllodocida | Polynoidae | Lepidonotus humilis | 3 | KP255273 |
| FTP_0289 | Annelida |  |  |  | 14 | KP254006 |
| FTP_0299 | Echinodermata | Ophiurida | Ophiactidae | Ophiactis savignyi | 19 | KP254102 |
| FTP_0309 | Echinodermata | Ophiurida | Amphiuridae | Amphipholis cf. squamata | 21 | KP254038 |
| FTP_0402 | Platyhelminthes |  |  |  | 28 | KP254462 |
| FTP_0480 | Arthropoda | Decapoda |  |  | 33 | KP254861 |
| FTP_0530 | Arthropoda | Isopoda | Sphaeromatidae | Cilicaea | 5 | KP254210 |
| FTP_0536 | Arthropoda | Decapoda | Hippolytidae |  | 7 | KP253998 |
