# Supplementary material for: Random sampling causes the low reproducibility of rare eukaryotic OTUs in Illumina COI metabarcoding
Source: PeerJ. 2017 Mar 22;5:e3006. doi: 10.7717/peerj.3006 (PMC5364921; doi:10.7717/peerj.3006)
Supplement: Figure S3 — We used a general time reversible nucleotide model with a proportion of invariant sites and among site rate heterogeneity modeled with a discrete gamma distribution (GTR +I +G) together with GARLI default settings, including stepwise-addition starting trees. Branch tips indicate the mean of identification of each OTU. Mock: OTU that matched the COI sequence of a species included in the mock sample; NCBI/BOLD: OTU that did not match a target OTU but had >98% similarity with a reference COI barcode in NCBI or BOLD; SAP, OTU that did not match a target OTU nor a reference COI barcode in NCBI or BOLD but could be confidently assigned to higher a taxonomic level using a Bayesian phylogenetic approach implemented in the Statistical Assignment Package (SAP); Unknown: OTU that could not be confidently identified to any taxonomic group using the three approaches detailed above. [file peerj-05-3006-s003.docx]

**Figure S1** Phylogenetic relationships between representative COI sequences (313bp) of 120 OTUs detected in the mock sample estimated using a Maximum Likelihood approach. We used a general time reversible nucleotide model with a proportion of invariant sites and among site rate heterogeneity modeled with a discrete gamma distribution (GTR+I+G) together with GARLI default settings, including stepwise-addition starting trees. Branch tips indicate the mean of identification of each OTU. Mock: OTU that matched the COI sequence of a species included in the mock sample; NCBI/BOLD: OTU that did not match a target OTU but had >98% similarity with a reference COI barcode in NCBI or BOLD; SAP: OTU that did not match a target OTU nor a reference COI barcode in NCBI or BOLD but could be confidently assigned to higher a taxonomic level using a Bayesian phylogenetic approach implemented in the Statistical Assignment Package (SAP); Unknown: OTU that could not be confidently identified to any taxonomic group using the three approaches detailed above.
