# Supplementary figures and images for: Random sampling causes the low reproducibility of rare eukaryotic OTUs in Illumina COI metabarcoding
Source: PeerJ. 2017 Mar 22;5:e3006. doi: 10.7717/peerj.3006 (PMC5364921; doi:10.7717/peerj.3006)

Jaccard dissimilarity

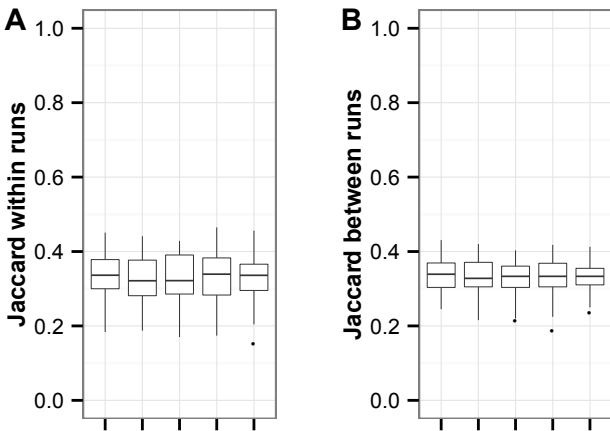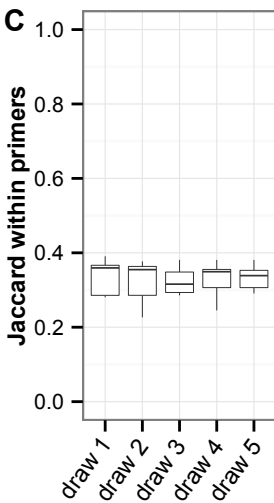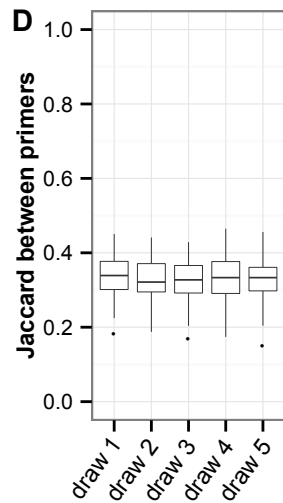

Rarefaction draws

Bray-Curtis dissimilarity

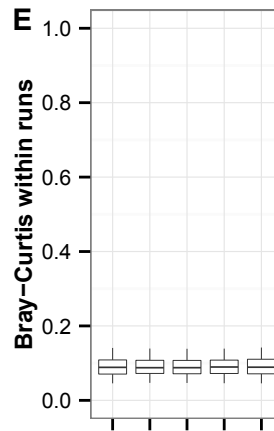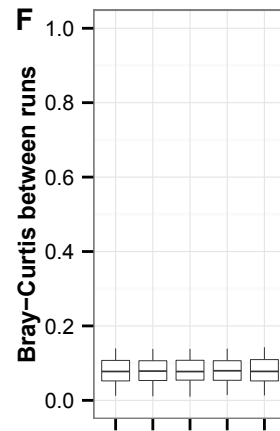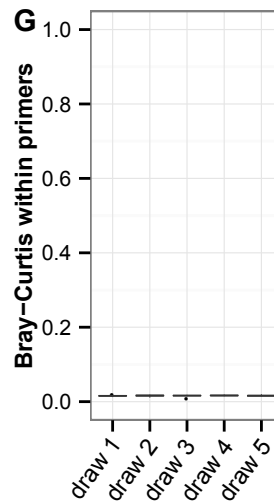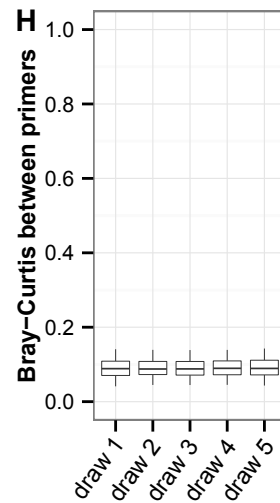

Rarefaction draws

Supplement: Figure S2 — The OTU table was rarefied five times down to the lowest number of sequences that a sample contained (45,609). See Table 2 for a summary of pairwise dissimilarity calculations. [file peerj-05-3006-s004.pdf]
